# Phenotypic Description of *Theobroma cacao* L. for Yield and Vigor Traits From 34 Hybrid Families in Costa Rica Based on the Genetic Basis of the Parental Population

**DOI:** 10.3389/fpls.2018.00808

**Published:** 2018-06-19

**Authors:** Guiliana M. Mustiga, Salvador A. Gezan, Wilbert Phillips-Mora, Adriana Arciniegas-Leal, Allan Mata-Quirós, Juan C. Motamayor

**Affiliations:** ^1^Mars Incorporated, Miami, FL, United States; ^2^School of Forest Resources and Conservation, University of Florida, Gainesville, FL, United States; ^3^Tropical Agricultural Research and Higher Education Center (CATIE), Turrialba, Costa Rica

**Keywords:** cacao, combining ability, heritability, heterosis, yield stability, genetic gains

## Abstract

A comprehensive understanding of the genetic basis of target traits in any crop is critical to design breeding strategies for the development and release of new improved varieties. In this study, 34 cacao families were evaluated for vigor and yield related traits over the course of 6 years in Costa Rica. Linear mixed models provided the variance components for the partitioning of additive and non-additive effects. Heritabilities of yield over time ranged from 0.085 to 0.576, from 0.127 to 0.399 for vigor, and 0.141 to 0.146 for disease resistance traits. Significant (*p* < 0.001) general combining abilities were observed for ICS-43 and LcTeen-37 with negative effect on average yield (−0.674, −0.690), respectively. Specific combining abilities for yield had significant (*p* < 0.001) positive effect from the cross GU-154-L x UF-273 Type 2 (0.703) and strong negative interaction between ICS-43 and LF-1 (−0.975). A weighted index was used to select the top performers while providing the corresponding genetic gains. At an 1% selection intensity, yield component gains ranged from 17.8 to 331.9%. Agronomic traits such as branch angle, trunk diameter and jorquette height had lower genetic gains and lower heritabilities. In addition, the parents in this study were genotyped with a 96-SNP marker off-typing set and a significant positive correlation of 0.39 (*p* = 0.019) was found between genetic distance and specific combining abilities for yield. Preliminary comparison of clonal parents vs. seedlings yield in the family with the highest SCA suggest for the first time presence of heterobeltiosis in cacao.

## Introduction

*Theobroma cacao* L is a diploid species with 10 pairs of chromosomes (2*n* = 2*x* = 20). It is an understory fruit-tree species from the Malvaceae family native to South America (Motamayor et al., 2002). As a cash crop, cacao is grown for its fruits, known as cacao pods, where its seeds (beans) are used in the making of chocolate, cocoa butter, and cosmetics. The dried and processed cacao beans are the basis of the confectionary industry and can be the main source of income for small holder farmers in some tropical regions. Cacao's world production of roughly 4 million tons is currently dominated by West Africa (72.4%) with Cote d'Ivoire as the main producer, followed by South and Central America (16.6%) and Asia Pacific (10%) (ICCO, 2017). Over the last 20 years, cacao production has increased as a result of larger planting areas, while yields have remained low, averaging ~300 kg/ha (Motamayor et al., 2008). In West Africa, cacao production has expanded more rapidly compared to other export-oriented crops, with 6.3 Mha of land allocated to growing cacao (Ordway et al., 2017).

Cacao faces multiple challenges such as market price fluctuations, the effect of old and new devastating diseases, use of low inputs and technological solutions and lack of improved certified planting material that would provide farmers sustained productivity and profitability. Cacao yields can be increased significantly using improved genetic materials and better agronomical practices. Breeding programs require a deeper understanding of the genetics controlling yield and disease traits. This is key for breeding new outstanding cultivars that will provide higher yields relative to planting density or area. The primary goal for most breeding programs at the present is to select genotypes that have improved yield, resistant to pests and diseases and better bean quality while preserving genetic diversity. Moreover, for cacao, efforts should be focused on increasing yields through more efficient partitioning of dry beans relative to vegetative growth, selecting for higher dry bean to vegetative biomass ratios is a key challenge (Enriquez, 1981; Paulin et al., 1993) to optimize land use. There's significant potential to break the high yield–high vigor correlation, in favor of high yield and low vigor, since current cacao varieties are only a few generations from the wild, leaving substantial genetic variation for both yield and vegetative growth (Yapp, 1992; Daymond et al., 2002). In addition, planting density is an important factor in determining the yield of cacao genotypes (Mooleedhar and Lauckner, 1990; Lockwood and Pang Thau Yin, 1996) which makes yield efficiency, the ratio of yield to planting area, which is heritable (Pang, 2006), an important trait to include in a breeding program.

A good and complete understanding of the genetic basis of target traits, in any crop, is critical to design integral breeding strategies and to implement the necessary steps for the development and release of new improved varieties. Detailed evaluations of field experiments for specific populations provide with a measure of the level of genetic control (i.e., heritability) of a given trait, the magnitude of partition of the genetic variance into additive and non-additive genetic effects, the type and amount of genetic correlation that exists between traits, and, in cases where there are multiple site experiments, the magnitude and dynamics of genotype-by-environment interactions. All of the above elements are required to understand the genetic basis of the traits of interest and they are essential to evaluate the potential outcomes in terms of future genetic gains of an operational breeding program.

Cacao breeding programs, with the implementation of recurrent selection from a diverse population began in the 1930s (Dias, 2001). Several experiments to evaluate genotypes had been established in many countries; however, published results related to genetic architecture of traits are limited. Some recent studies have reported interesting high to moderate narrow-sense heritabilities for traits related to yield (0.37–0.64) and disease traits (0.03–0.16) (DuVal et al., 2017); while other studies have reported moderate to low broad-sense heritabilities for yield traits (0.06–0.23) and plant vigor (0.02–0.14) (Ofori et al., 2016). In addition, partitioning the genetic signal into an additive (i.e., general combining ability) and a dominant (i.e., specific combining ability) effect has been reported (Dias and Kageyama, 1995; Cervantes-martinez et al., 2006; DuVal et al., 2017), with a strong contribution of the dominance, confirming what is known from this crop, where high levels of heterosis, among specific genetic groups, are found due to genetically diverse origins (Dias, 2001). The above results apply to specific populations and environments, and therefore, they need to be determined for each breeding program individually.

The main objective of this study is to contribute to a better understanding of the phenotypic variation of target breeding traits in cacao through the evaluation of families from a long-term field experiment established in Costa Rica and to provide insight into the genetic basis of target traits through the evaluation of the parental population planted in a clonal trial. At the same time, these trails are part of a breeding program aiming at ultimately delivering new clones to farmers. Indeed, farmers have limited access to improved cacao cultivars and breeding is necessary to develop the planting material farmers need. To improve yields, farmers require cultivars that have low vigor, high yield, and disease resistance so that land and pesticide use becomes more efficient while increasing farmers' income. The trial was the result of a factorial crossing scheme measured for 6 years in mature trees. The specific objectives of this work include: (1) to calculate narrow-sense heritability and dominance ratio for various key traits, (2) to estimate general and specific combining ability effects and their relationships with genetic distance, (3) to determine additive and dominant correlation between contrasting traits, (4) to estimate genetic correlations for yield between different measurement years, (5) to explore response to early or late selection on yield, and (6) to calculate genetic gains for different selection intensities. Special emphasis was given to the understanding of the dynamics of cocoa yield in regard to time and tree age.

## Materials and methods

### Genetic material

#### L7 seedling trial

The L7 field trial was established on the Atlantic coast of Matina, Costa Rica (10° 06′ latitude N, 83° 23′ longitude W, 40 m a.s.l) in mid-2003 at the CATIE's “La Lola” experimental farm under a randomized complete-block-design (RCBD) spanning an area of 2.6 ha at a density of 3 × 3 m. The layout considered five replications, each with four plots of each family, and plots were formed by four trees. Thus, there is a total of 80 plants per family and 2,720 trees for the experiment, plus additional border trees, with Banana plants (*Musa* spp.), planted at a spacing of 6 × 6 m, and immortelle trees (*Erythrina poeppigiana*), planted at 12 × 12 m, were added to the experiment as temporary and permanent shade, respectively. Cacao trees were given structural pruning at the beginning of the trial and periodic maintenance pruning thereafter. Every 3 months, 150 g of granular fertilizer (formula 18-5-15-6-0.3-7) were applied. Manual weed control was carried out every 2 months, complemented by two annual directed applications of paraquat herbicide (0.2 kg/ha). No disease control was carried out in the trial other than cutting diseased fruits at the time of the monthly evaluations, which were then left on the soil without applying any treatment. The L7 trial consists of 34 full-sib families that originated from a full factorial crossing scheme based on 12 parents (5 females and 7 males, where only one cross was missing) (Supplementary Table S1). The parents considered in the crossing scheme come from diverse genetic backgrounds and they were selected according to different contrasting breeding characteristics, mainly resistance to frosty pod (UF-273 Type I, ICS-43, IMC-60, and PA-169), black pod (PA-150 and GU-154 L), Witches' broom (Lc-Teen-37), and Ceratocystis wilt (IMC-67), but other relevant characteristics were considered, such as, dwarfism for improved planting density (Santa Clara-3 and LF-1) and industrial flavor quality (Criollo-27).

#### L10 clonal trial

The parents of the crosses in L7 were planted as clones in the L10 field, in the same experimental farm “La Lola” at CATIE. This trial was planted in July-December 2005 involved the 12 parents in L7 and two additional clones for a total of 14 clones: Lc-Teen-37, PA-150, GU 154-L, IMC-67, IMC-60, Criollo-27, ICS-43, LF-1, UF-273 Type 1, UF-273 Type 2, Santa Clara-3, PA-169, CATIE-R89, and Tree 40 from the cross “CC-252 × SCA-6”. An RCBD with 14 treatments (clones), four replications and 8 plants per replication and clone was used. Cacao trees were established at 3 × 3 m. Banana plants and immortelle trees were planted at 9 × 9 m as temporary and permanent shade species, respectively.

#### Traits measured

Phenotypic data was collected monthly from June 2004 to May 2010, corresponding to tree ages 2–7. The traits measured monthly were: fresh bean weight (FBW, g), number of total pods (TP), number of healthy pods (HP), number of pods infected with Monila (*Moniliophthora roreri*), a clonal fungal pathogen (Díaz-Valderrama and Aime, 2016) that causes cacao beans inside the pods to rot (MO), and number of pods infected with black pod, *Phytophthora* spp., a plant-pathogenic oomycetes (Fulton, 1989) that is the most widespread pathogen and comes in several forms and causes damage to trunks, branches and pods (PHY). In addition, trunk diameter was measured at 30 cm above ground at age 5 (TD5, cm), and jorquette height (JH2, cm) was measured at age 2. Branch angle (BA2, °) was measured 17 months after planting. Total yield (Yield, kg/ha) was estimated as 0.4 × FBW (fresh bean weight measured in grams)^*^1111.11(plant density)/1,000 for each age (Y2, Y3, Y4, Y5, Y6, and Y7), also an average yield (Ym) and its standard deviation (Ysd) were calculated across these years. Additionally, precocious yield was defined as the average yield for the ages 2 and 3 (Yinit), and full production yield was defined as the average yield between ages 4 and 7 (Ylate). Pod index (PI), defined as the number of healthy pods needed to obtain 1 kg of healthy dry bean weight, was calculated as PI = HP^*^1000/FWB^*^0.4, where 0.4 is the FBW to dry bean weight conversion factor (Somarriba and Beer, 2011). Proportion of healthy (%HP), Monilia (%MO) and Phytophthora infected pods (%PHY) were also calculated as the ratio of their respective cumulative counts from all years divided by the sum of total number of harvested pods. Finally, yield efficiency at age 5 (YE5, kg/ha/cm) was calculated as the ratio between yield and trunk diameter at age 5 as YE5 = Y5/TD5. Further descriptions and summary statistics of these traits are presented in Table 1.

**Table 1:** Description and summary statistics of available traits from the L7 population established in Costa Rica (10° 06′ latitude N, 83° 23′ longitude W, 40 m a.s.l) in mid-2003 at CATIE's “La Lola” experimental farm.

**Trait**	**Trait description**	**Age**	**Units**	**Transformation**	**Favorable direction**	**Mean**	**SD**	**Range**
HP	Healthy pods	2–7	count	–	high	5.6	5.4	0.0–55.0
MO*	Monilia pods	2–7	count	–	low	1.7	2.0	0.0–21.7
PHY*	Phytophthora pods	2–7	count	–	low	0.0	0.2	0.0–4.5
FWB	Fresh bean weight	2–7	grams/yr	–	high	580.2	550.6	0.0–5,459.5
TP	Total pods	2–7	count	ln(x+10)	high	7.3	6.7	0.0–59.5
%HP	Proportion of healthy pods	2–7	percent	–	high	76.5	17.4	0.0–100
%MO	Proportion Monilia pods	2–7	percent	logit(x+0.025)	low	23.2	17.2	0.0–100
%PHY*	Proportion Phytophthora	2–7	percent	–	low	0.3	1.8	0.0–28.1
PI	Pod index	2–7	index	ln(x+10)	low	26.6	13.6	0.0–229.5
JH2	Jorquette height age 2	2	cm	–	low	108.9	25.6	0.0–221.4
BA2	Branch angle age 2	2	angle	–	low	45.3	10.9	0.0–78.0
TD5	Trunk diameter age 5	5	cm	–	low	8.1	1.6	0.0–14.2
YE5	Yield efficiency age 5	5	kg/ha/yr/cm	ln(x+10)	high	38.4	41.7	0.0–376.3
Y2	Yield age 2	2	kg/ha/yr	ln(x+10)	high	6.2	39.8	0.0–746.6
Y3	Yield age 3	3	kg/ha/yr	ln(x+10)	high	101.4	215.1	0.0–2,986.4
Y4	Yield age 4	4	kg/ha/yr	ln(x+10)	high	343.4	453.3	0.0–5,423.6
Y5	Yield age 4	5	kg/ha/yr	ln(x+10)	high	328.8	392.2	0.0–3,797.0
Y6	Yield age 6	6	kg/ha/yr	ln(x+10)	high	396.2	447.1	0.0–3,150.8
Y7	Yield age 7	7	kg/ha/yr	ln(x+10)	high	371.6	477.6	0.0–4,436.9
Ym	Mean yield ages 2–7	2–7	kg/ha/yr	ln(x+10)	high	257.8	244.7	0.0–2,426.2
Ysd	Yield standard deviation ages 2–7	2–7	kg/ha/yr	ln(x+10)	low	265.4	226.1	0.0–1,900.6
Yinit	Initial mean yield ages 2–3	2, 3	kg/ha/yr	ln(x+10)	high	53.8	114.3	0.0–1,493.2
Ylate	Late mean yield ages 4–7	4–7	kg/ha/yr	ln(x+10)	high	359.9	342.7	0.0–3,428.2

* *Traits not included in analyses*.

### Statistical analyses

For the statistical analyses all traits described earlier where evaluated except for some count traits (HP, MO, and PHY), which are weakly informative for assessing incidence of disease. In addition, %PHY was not considered as the level of Phytophthora in this trial was very low (~0.5%). Note also that with low levels of PHY, then %HP ~ 100%–%MO; hence, there is a high negative correlation between %HP and %MO. Natural logarithm and logit transformations were implemented in some traits to approximate normality of residuals (further details in Table 1).

For each the traits previously specified, the following linear mixed animal model was fitted:

$$\begin{array}{l} y=\mu 1+Xb+Z_{1}a+Z_{2}f+Z_{3}p+e \end{array}$$

where ***y***is the vector of response variables; μ is the overall mean; *b* is the fixed vector of replicate effects; *a* is the random effect of individuals additive effects, with multivariate normal distribution with mean *0* and variance $\sigma_{\text{a}}^{2}$*A*, i.e., *a*~MVN(*0*, $\sigma_{\text{a}}^{2}$*A*); *f* is the random vector of family effects, with *f*~MVN(*0*, $\sigma_{\text{f}}^{2}$*I*_*f*_); *p* is the random vector of plot effects, with *p*~MVN(*0*, $\sigma_{\text{p}}^{2}$*I*_*p*_); and *e* is the random vector of errors, with *e*~MVN(*0*, $\sigma_{\text{e}}^{2}$*I*_*e*_). The matrix *A* is the numerator relationship matrix obtained from pedigree, the *I* matrix is an identity matrix of its corresponding size, *1* is a vector of ones, and the incidence matrices for the respective effects are represented by *X, Z*_1_, *Z*_2_, and *Z*_3_.

After fitting the above model, estimated general combining ability (GCA, which is $\frac{1}{2}$ of the additive term) and specific combining ability (SCA, which is identical to the family term) effects were obtained for each parent and parental combination, and the variance component estimates were used to calculate narrow-sense heritability (*h*^2^) and dominance ratio (*d*^2^) (their standard errors were obtained by approximation using the Delta method). These expressions corresponded to:

$$\begin{array}{l} h^{2}=\sigma_{a}^{2}/\left(\sigma_{a}^{2}+\sigma_{f}^{2}+\sigma_{p}^{2}+\sigma_{e}^{2}\right)\\ d^{2}={4\sigma }_{f}^{2}/\left(\sigma_{a}^{2}+\sigma_{f}^{2}+\sigma_{p}^{2}+\sigma_{e}^{2}\right) \end{array}$$

In addition, bivariate analyses were performed by fitting a linear model that considered two traits simultaneously in order to estimate additive and dominance trait-to-trait correlations. The general linear mixed model fitted corresponded to:

$$\begin{array}{l} y=X_{1}t+X_{2}bt+Z_{1}at+Z_{2}ft+Z_{3}pt+e \end{array}$$

where *y* is the vector of the two stacked response variables; *t* is the fixed effect of trait; *bt* is the fixed vector of replicate effects within trait; *at* is the random effect of additive effects within trait, with *at*~MVN(*0, A*⊗*G*_*a*_); *ft* is the random vector of family effects within trait, with *ft*~MVN(*0, I*_*f*_⊗*G*_*f*_); *pt* is the random vector of plot effects within trait, with *pt*~MVN(*0, I*_*t*_⊗*D*_*p*_); and *e* is the random vector of errors, with *e*~MVN(*0, I*_*e*_⊗*D*_*e*_). The 2 × 2 matrices *G*_*a*_ and *G*_*f*_ are additive and dominant variance-covariance matrices of structure CORUH between traits, respectively; *D* are 2 × 2 diagonal matrices, and the other terms were previously defined. The above bivariate model provides directly with an estimate of the additive and dominant trait-to-trait genetic correlations.

All linear mixed models were fitted with the statistical software R (R Core Team, 2015) using the library ASReml-R v.3 (Butler et al., 2009) which uses restricted maximum likelihood methods to estimate variance components. In all cases, diagnostic plots were assessed for normality and to detect potential outliers.

### Genotyping of L10

In order to improve analyses based on pedigree and in efforts to gain more information about the 34 families in this trial, the genotyping of L10 (parents of L7) was done on a representative clone from each replication using the 96 SNP off-typing marker described in DuVal et al. (2017). A total of four representative genotypes of the parental clones were compared. The four clonal replications of GU-154L, IMC-60, IMC-67, PA-169, UF-273 Type I, and UF-273 Type 2 had identical genotypes for each of the four leaf samples. The remaining six parents (Criollo-27, ICS-43, Lc-Teen-37, LF-1, PA-150, and Santa Clara-3) had differing genotypes (off-types) in the four leaf samples. To calculate genetic distances among representative genotypes of each parental clone, a single genotype was chosen for those clones showing off-types. Representative genotypes were chosen by comparing each different genotype of a same clone with reference genotypes representing the genetic group (Motamayor et al., 2008) to which the original clone belongs to as described in the International Cocoa Germplasm Database (ICGD) database. Genetic distances (GD) between the parents and with reference genotypes were calculated with the function dist.gene from the “ape” package in the R software (Paradis et al., 2004; R Core Team, 2015). The genetic distance for each set were compared to the matrix of SCAs for Ym given what is known about the positive relationship between GD, yield and heterosis in cacao and in other crops (Dias et al., 2003). The L10 genotypes used to calculate the correlation between GD and SCA are represented in Supplementary Table S2 along with percentage of heterozigosity based on the 96 SNP markers and the coefficient of membership to each of the 10 cacao ancestral groups previously described (Motamayor et al., 2008). A supervised admixture analysis was performed with Bayesian modeling from the Structure program (Pritchard et al., 2000) with 2,000 reference genotypes simulated with 40 SNP markers and the Bayesian clustering approach described in Olasupo et al. (2018). The admixture analysis included 200 individuals from the 2,000 simulated references with over 85% proportion ancestry per each of the 10 cacao ancestral groups, *K* = 10 clusters, a burn-in of 100,000 iterations and 200,000 replicates.

### Genetics gains and response from selections

In order to evaluate response to selection, best linear unbiased prediction (BLUP) values from both hybrids and parents were obtained from the single-trait animal model (Lynch and Walsh, 1998; Gilmour et al., 2009). Individuals were ranked from most favorable to least favorable and the average values (i.e., genetic gains) from the top 1% (*n* = 27) and 5% (*n* = 136) performing hybrids were calculated. Genetic gains were expressed as: (i) the percent increase of the 1 or 5% selection intensities over the overall mean in L7 **(Table 3)**, (ii) the gain (based on phenotypic data) of the entire L7 progeny population relative to the parents planted as clones in the L10 clonal trial (Supplementary Table S7), (iii) the gain (based on breeding values from animal model) of the top 1 and 5% trees in L7 relative to the predicted value of the parents in L7 (Supplementary Table S8), and (iv) the grand mean of the L7 progeny relative to the predicted value of the parents in L7 (Supplementary Table S8), and (v) the genetic gain of the top 1% and 5% L7 progeny for yield traits (Y2-Y7, Ym, Yinit, Ylate) relative to the overall L7 progeny yield mean (Ym) (Supplementary Table S9). Traits selected for high values were Y1-Y7, Ym, YE5 and TP, and traits selected for low values corresponded to PI, %MO, JH2, and TD5.

### Selection for yield

To obtain further understanding of cocoa yield in regard to tree age and its dynamics, several measurements (Y2, Y3, Y4, Y5, Y6, and Y7) and definitions of yield (Ym, Ysd, Yinit, and Ylater) were explored. Heritabilites and dominance ratios were obtained from the single-site model for all traits, together with genetic additive and dominant correlations between traits based on the above definitions from the bivariate models. Of interest is the genetic association between precocious yield (Yinit) and full production yield (Ylater) in order to assist with response to early selection. Additionally, predictive performance of parents (GCA) was compared across these traits.

### Ideotype characterization

The centered and scaled predicted values (z-scores) of the individuals and parents from the single trait analyses were ranked and used in a linear combination (with weights assigned to each trait in order to combine them in an optimal form). The rankings of the z-scores were first obtained for each individual by trait, where the low ranks are favorable and the high ranks are least favorable. Percent monilia infected pods (%MO), branch angle (BA2), pod index (PI), jorquette height (JH2), and trunk diameter (TD5) were ranked in ascending order (where the top hybrids showed the lower values). Average yield across years (Ym) and Yield efficiency at age 5 (YE5) were ranked in descending order (where the hybrids with the higher values are ranked first). All rankings were adjusted for ties by averaging those equally ranked. The weights chosen to construct the index were based on heritability and the known economic importance of each trait. The best individuals correspond to those whose index value (based on the rank for each trait) is the lowest. The expression for the index is denoted as:

$$\begin{array}{l} Index_{i}=Z_{\left(ixk\right)}w_{\left(kx1\right)} \end{array}$$

where, *Z* is a matrix of the ranked z-scores for individual *i* for each of the *k* traits, *w* is a vector of length *k* for the weights (**Table 5**) assigned to each trait.

## Results

Phenotypic measurements for the L7 population (presented in Table 1) show a wide range of responses for most traits. Figure 2 presents the phenotypic correlations between the traits, those among the highest were between Ym and YE5 (0.69) and Ym with TD5 (0.55). TD5 and JH2 have a correlation of 0.27, and for JH2 with BA2 is 0.12. Small correlations were found for pod index (PI) with all the traits, where the highest was with Ym (0.19). The proportion of monilia infected pods (%MO) was highest correlated with Ym (0.17) and JH2 (0.12). The mean proportion of healthy pods for the entire trial corresponded to 76.5%, where the majority of the diseased pods (23.2%) was affected by Monilia, and only a small proportion (0.3%) was affected by Phytophthora. For yield, the means over time increased considerably at ages 2 and 3, and reached a plateau at age 4. Nevertheless, large amounts of variability were observed for annual yield with coefficient of variation values ranging from 113 to 642% (largest for Y2 and Y3), which is due to the high proportion of trees with no pod production (with 80% for Y2, 46% for Y3, and then dropping to 15% by Y7).

### Single-trait analyses

A summary of estimated heritabilities and dominance ratio values are presented in Table 2. JH2 had one of the highest heritabilities with a value of 0.399. Low heritabilities were found for TD5, PI, %HP, %MO, and BA2, which ranged from 0.127 to 0.205. The heritability for %MO was low with a value of 0.141, reflecting the low level of genetic control for this trait.

**Table 2 T2:** Narrow-sense heritability (*h*^2^) and dominance ratio (*d*^2^) for evaluated traits calculated from the single-trait analysis.

**Trait**	***h*^2^**	**SE (*h*^2^)**	***d*^2^**	**SE (*d*^2^)**
JH2	0.399	0.157	0.100	0.048
TD5	0.205	0.116	0.314	0.111
BA2	0.127	0.069	0.111	0.051
Y2	0.085	0.049	0.057	0.038
Y3	0.341	0.153	0.265	0.097
Y4	0.379	0.166	0.304	0.110
Y5	0.339	0.156	0.306	0.110
Y6	0.323	0.146	0.238	0.090
Y7	0.339	0.151	0.258	0.095
Ym	0.576	0.223	0.484	0.167
PI	0.158	0.113	0.459	0.152
TP	0.419	0.188	0.426	0.147
HP	0.426	0.189	0.433	0.148
%MO	0.141	0.075	0.115	0.055
%HP	0.146	0.076	0.112	0.054
Yinit	0.352	0.157	0.265	0.098
Ylate	0.542	0.218	0.497	0.170
Ysd	0.459	0.208	0.573	0.189
YE5	0.292	0.137	0.237	0.090

*Standard errors (SE) were approximated by the Delta method. Definition of traits are presented in Table 1*.

It was also noted that heritabilities for traits related to yield were high ranging from 0.419 for TP to 0.576 for Ym, with the exception of Y2 that presented a value of 0.085. Heritabilities increase after age 3 and then become relatively stable over the later measurements. Ysd presents a heritability of 0.459, which can allow for selection of genotypes that have low variability on pod production. Interestingly, higher heritabilities were found for late years (0.542), in contrast to early years (0.352).

In general, the L7 population has overall high dominance ratio, likely due to the high genetic diversity of the parents and heteroric effects. Dominance ratio (*d*^2^) is highest for Ym, Ysd, Ylate, PI, and TP (0.426–0.573). Moderate *d*^2^ levels are found for TD5, Y3 to Y7 and YE5 with *d*^2^-values ranging from 0.237 to 0.314. Low dominance ratios were observed for %MO, %HP, BA2, JH2, and Y2 (0.057–0.115). It is important to note that PI, TD5, Ysd, TP, and Ylate showed a larger dominance ratio than heritability (i.e., *d*^2^/*h*^2^ > 1).

### Bivariate analyses

Several bivariate models were fitted to obtain genetic correlation estimates between pairs of selected traits for both additive and dominant effects (Figures 4–7; Supplementary Tables S3, S4). The correlation estimates between average yield (Ym) and vigor traits (TD5, BA2, and JH2) are moderate to high in absolute value (0.783, −0.637, 0.509, respectively) reflecting the importance of observing plant vigor traits to improve overall yield. Interestingly, additive effects of BA2 are negatively and strongly related to Ym (−0.637), indicating that plants with lower angles (having a more upright position) present higher yields. TD5 is also strongly negatively related to BA2 (−0.794), which suggest that plants with large diameter tend to have small branch angle (more erect plant architecture), and similarly the correlation between TD5 and Ym is strong and positive (0.783). In contrast, there is no additive correlation between BA2 and JH2 (0.01). As expected, there is a high correlation between %HP and %MO but this is non-informative in this case given that Monilia infected pods and healthy pods are complements of each other. PI and %HP (and correspondingly, %MO) have a high negative correlation (−0.670) suggesting that tolerant hybrids tend to develop better bean weight to pod weight values. As expected, Ym and TP are strongly positively correlated (0.973). YE5 is highly correlated, in absolute values, with Ym (0.982), PI (−0.835), and TP (0.953). In addition, YE5 has almost null correlations with %HP (0.04) and %MO (−0.05).

The additive genetic correlations between yield traits at different ages (Y2–Y7), Ylate (Y4–Y7), and Yinit (Y2–Y3) are all positive and relatively high with values ranging from 0.778 to 0.999. As expected, higher correlations were found between close ages. However, Y2 presents the lowest heritability and also the lowest dominance correlation with other traits; for example, the dominance correlation between Y2 and Y3 is 0.683, and close to zero for Ym, as well as for the later years Y6 and Y7. The additive correlation between Ylate and Yinit was 0.933, indicating that, regardless of the differences in heritabilities, these two traits provide with similar ranking of genotypes. The genetic additive correlations between Y2 with Ym is 0.835, and Y3 to Ym is 0.935; hence, there is better genetic agreement from Y3 for indirect selection of high overall yielding genotypes.

The estimates of dominance genetic correlation showed, generally, weaker associations, in absolute value, than the majority of the additive correlations, but they follow similar trends (Figures 6, 7; Supplementary Tables S3, S4). However, in some cases these were considerably high, particularly with some of the yield traits, this is likely to be associated with large standard errors associated with their estimation.

### Genetic gains and genetic effects

Two selection intensities were considered for the calculation of genetic gains based on individual best linear unbiased predictor values: 1% (*n* = 27), and 5% (*n* = 136). As expected, given that cacao is in its early stages of domestication (Motamayor et al., 2008), large genetic gains can be achieved for yield related traits (Table 3). For any of the years Y3 to Y7, large genetic gains can be achieved as these are the traits with the highest heritability estimates. At 5% selection intensity, Y4 provides 146% of genetic gain, 240% for Y3 and 197% for Y2. Similarly, Ysd has a high genetic gain (75.6%). Vigor traits (JH2, BA2, and TD5) have lower genetic gains ranging from 12 to 20% at 5% selection intensity. The lowest gains are attributed to %TD and %HP (9.4 and 10.6%, respectively).

**Table 3:** Genetic gains.

**Trait**	**Favorable direction**	**Top 1%**		**Top 5%**		**all**
		**Mean**	**Gain %**	**Mean**	**Gain %**	**Mean**
JH2	Low	84.6	21.9	86.6	20.1	108.4
TD5	Low	7.2	11.1	7.3	9.4	8.1
BA2	Low	38.8	14.1	39.8	11.9	45.2
PI	Low	20.7	17.8	21.3	15.3	25.2
%MO	Low	0.1	47.1	0.1	43.2	0.2
Ysd	Low	29.9	84.7	47.2	75.8	195
YE5	High	57.8	127.4	50.8	99.9	25.4
Y2	High	4.5	284.5	3.5	197.1	1.2
Y3	High	111	331.9	87.4	240.2	25.7
Y4	High	415.1	185.5	358.1	146.3	145.4
Y5	High	411.4	167.0	351.9	128.4	154.1
Y6	High	478.6	147.8	420.5	117.7	193.2
Y7	High	440.7	165.6	379.5	128.7	166
Ym	High	571.3	207.7	454	144.6	185.6
Yinit	High	70.6	305.3	56.8	225.7	17.4
Ylate	High	737.7	193.5	595	136.7	251.3
TP	High	13.5	113.6	11.6	82.6	6.3
HP	High	11.4	133.5	9.4	90.9	4.9
%HP	High	0.9	11.5	0.9	10.6	0.8

*Individual progeny were ranked from most favorable to least favorable and the average values (i.e., genetic gains) from the top 1% (n = 27) and 5% (n = 136) performing hybrids trees were calculated. Genetic gains were expressed as the percent increase of the 1% or 5% selection intensities over the overall mean. Traits selected for high values were Y1-Y7, Ym, YE5, HP, and TP, and traits selected for low values corresponded to PI, %MO, JH2, and TD5. Definition of traits are presented in Table 1*.

The estimation of GCA and SCA values for each of the parents and hybrid crosses are presented in Table 4. Some important differences between parents for most traits are noted. For example, parent PA-169 presents low levels of Monilia (%MO), and the hybrids from IMC-60 and LF-1 present very high levels of %MO. Other traits show relevant SCA effects, which reflects the high values of dominance ratio reported earlier that were found for this population.

**Table 4:** General and Specific Combining (GCA) abilities for parents in L7 for average yield (Ym), proportion of monilia infected pods (%MO), branch angle (BA2), total pods (TP), proportion of healthy pods (%HP), for jorquette height at 12 months (JH), trunk diameter at age 5 (TD5), and yield efficiency at age 5 (YE5) based on single-trait model fit.

**Trait**	**f/m**	**Criollo-27**	**GU-154-L**	**IMC-67**	**LcTeen-37**	**LF-1**	**PA-150**	**Santa Clara 3**	**GCA**
Ym	ICS-43	–	−0.222	0.453	−0.196	−0.975***	0.289	0.085	−0.674**
	IMC-60	–	–	−0.198	−0.067	0.35	−0.166	0.399	0.378
	PA-169	−0.179	−0.154	0.43	−0.169	−0.071	0.189	0.146	0.23
	UF-273(T1)	0.27	0.001	−0.208	0.192	0.449	−0.21	−0.375	0.141
	UF-273(T2)	0.147	0.703**	−0.272	−0.34	−0.075	0.005	−0.229	−0.075
	GCA	0.283	0.39	0.244	−0.690**	−0.385	0.127	0.03	
%MO	ICS-43	–	−0.051	0.128	−0.092	−0.271	0.141	0.137	−0.011
	IMC-60	–	–	−0.093	0.118	0.133	0.106	−0.084	0.221
	PA-169	0.202	−0.116	−0.189	−0.033	0.021	0.004	−0.163	−0.338**
	UF-273(T1)	−0.077	0.029	−0.018	0.205	0.068	−0.199	−0.05	−0.052
	UF-273(T2)	0.055	−0.014	0.022	−0.239	0.195	−0.019	0.145	0.179
	GCA	0.221	−0.186	−0.185	−0.05	0.178	0.04	−0.018	
BA2	ICS-43	–	0.622	−0.922	1.306	1.457	−0.037	−0.884	1.759
	IMC-60	–	–	1.573	0.525	−1.74	2.286	−1.27	1.566
	PA-169	−1.605	−0.628	1.738	−2.730*	0.962	1.192	0.257	−0.929
	UF-273 (T1)	−1.449	−1.018	−1.021	1.246	−0.285	−1.055	1.598	−2.263*
	UF-273(T2)	−0.055	0.92	−1.224	1.52	0.145	−1.515	0.093	−0.133
	GCA	−3.547**	−0.12	0.165	2.131	0.614	0.992	−0.235	
TP	ICS-43	–	−0.112	0.056	−0.007	−0.149*	0.074	−0.04	−0.177**
	IMC-60	–	–	−0.052	−0.051	0.036	−0.017	0.182**	0.097
	PA-169	−0.044	−0.081	0.132*	−0.031	−0.074	0.076	0.039	0.017
	UF-273(T1)	0.12	0.033	−0.039	0.007	0.119	−0.092	−0.095	0.052
	UF-273(T2)	0.04	0.253***	−0.09	−0.101	−0.002	−0.02	−0.07	0.011
	GCA	0.115	0.091	0.007	−0.180**	−0.069	0.021	0.015	
%HP	ICS-43	–	0.063	−0.141	0.093	0.253	−0.145	−0.123	0.001
	IMC-60	–	–	0.098	−0.108	−0.13	−0.12	0.081	−0.232
	PA-169	−0.195	0.111	0.191	0.025	−0.018	−0.003	0.16	0.350**
	UF-273(T1)	0.07	−0.031	0.011	−0.207	−0.054	0.205	0.048	0.056
	UF-273(T2)	−0.05	0.004	−0.021	0.242	−0.192	0.027	−0.146	−0.174
	GCA	−0.227	0.191	0.18	0.06	−0.183	−0.047	0.027	
JH	ICS-43	–	0.71	−3.201	4.894	−2.282	−2.609	−0.281	−11.011**
	IMC-60	–	–	−2.374	−6.776*	2.612	2.388	4.77	2.46
	PA-169	−3.257	4.728	0.838	−2.519	1.106	2.372	−0.848	9.623*
	UF-273(T1)	2.617	−2.514	0.91	2.691	1.57	−3.756	−1.152	1.455
	UF-273(T2)	−2.322	0.967	3.291	0.773	−2.288	0.979	−2.036	−2.527
	GCA	−11.774**	15.469***	−2.135	−3.728	2.855	−2.492	1.804	
TD5	ICS-43	–	−0.021	0.228	−0.209	−0.913**	0.318	0.1	−0.325
	IMC-60	–	–	0.047	−0.654*	0.057	0.081	0.396	−0.047
	PA-169	0.074	0.278	0.538*	−0.526	−0.164	0.193	0.204	0.39
	UF-273(T1)	−0.072	0.205	−0.125	0.504	0.386	−0.34	−0.474	0.055
	UF-273(T2)	0.099	0.566*	−0.743**	0.278	0.089	−0.354	−0.048	−0.073
	GCA	0.066	0.672**	−0.035	−0.396	−0.356	−0.066	0.116	
YE5	ICS-43	–	0.058	0.132	−0.028	−0.483***	0.096	−0.028	−0.325*
	IMC-60	–	–	−0.163	−0.207	0.390**	−0.04	0.25	0.295*
	PA-169	−0.126	−0.264	0.266	0.062	−0.003	0.087	0.047	0.089
	UF-273 (T1)	0.175	0.012	0.016	0.022	−0.109	−0.026	−0.102	−0.014
	UF-273 (T2)	0.138	0.278	−0.107	−0.194	0.061	−0.016	−0.196	−0.046
	GCA	0.242	0.108	0.185	−0.444**	−0.185	0.131	−0.037	

*** p < 0.001;

** p < 0.01;

* *p < 0.05*.

### Selection for yield

As indicated earlier, the highest heritability value was observed for Ym (0.576), followed closely by Ylate (0.542). The dominance ratio for the yield traits are close to their heritability estimates, indicating high levels of non-additive genetic variance. The dominance correlation between Yinit against Ylate is 0.345, indicating different behavior for ages 2–3 (juvenility period) compared to the later years. However, the additive correlation between Yinit and Ylate is 0.933, and at Y4 at age 4, there is very strong correlations with ages 5, 6, and 7. This suggests that Y4 can be used for early selection, which also has a strong correlation of 0.946 with Ym.

The variability of yield production, measured by the standard deviation of yield over the measured ages (Ysd) provided with a high heritability of 0.459 (Table 2). Additionally, there is high positive additive and dominance correlation between Ym and Ysd (0.999 and 0.981, respectively). This indicates that genotypes with high yield tend to have higher variability on their cocoa production and may suggest the need of trees to physiological rest after a high productive season as observed in other crops (Cannell, 1985).

**Figure 1:**
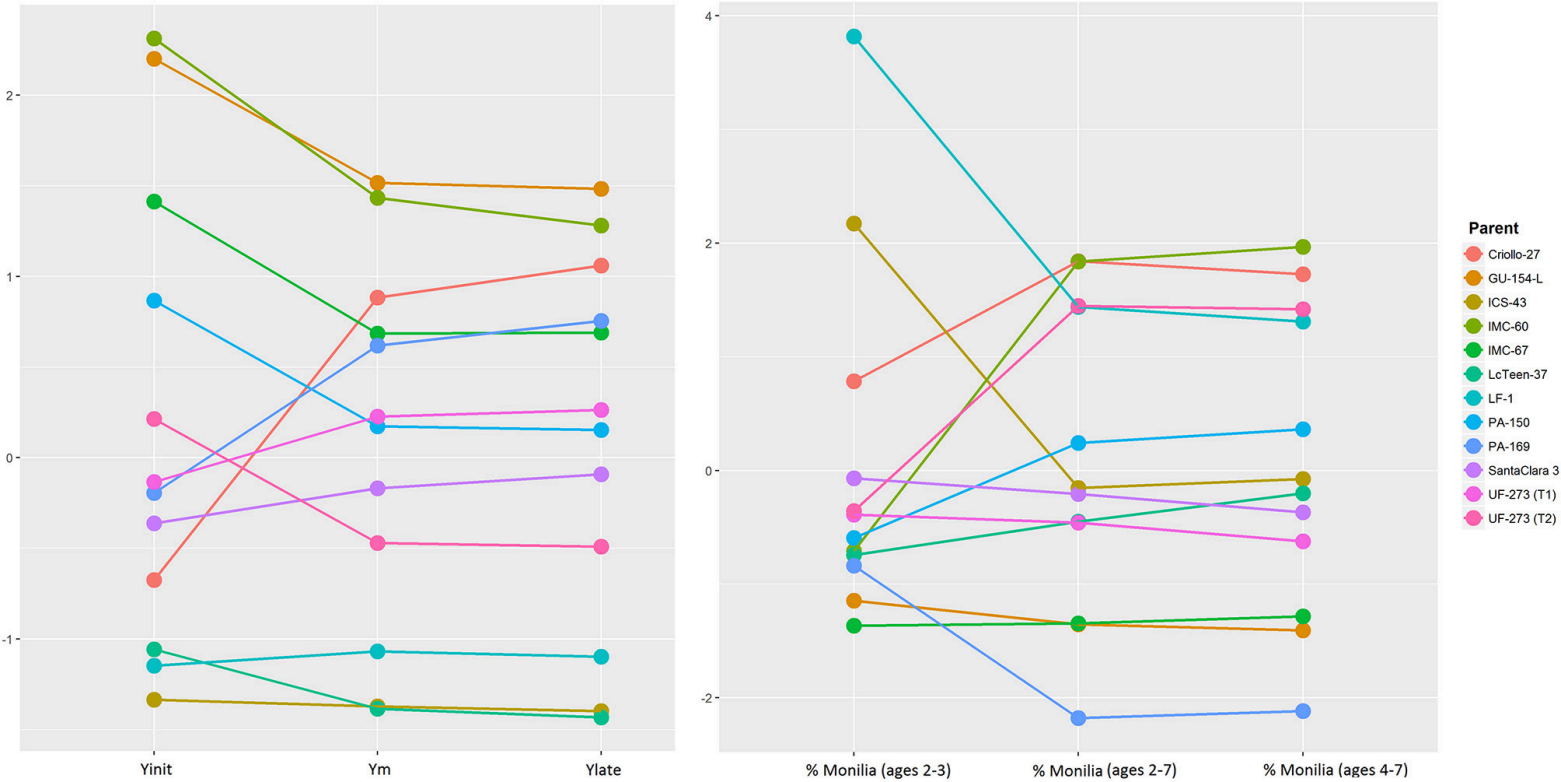
**Left**: Predicted values, and changes in ranking for different measures of cocoa yield: initial mean yield for ages 2, 3 (Yint) mean yield for ages 2–7 (Ym) and late mean yield for ages 4–7 (Ylate). **Right**: Predicted values and changes in ranking for percentage of Monilia infected pods for the same time points as yield. Further details of these traits are presented in Table 1.

Figure 1 presents the additive effects of the parents for Yinit, Ylate, and Ym from the standardized predicted values. Parents that are consistently high in rank relative to GCA effects of the other parents for the three traits are GU-154-L, IMC-60, IMC-67, and PA-169. Criollo-27 does not rank high for early yield; however, its rank changes to become one of the top parents for both late yield and average yield (in terms of additive effects). The consistently low performers for these three traits (Yinit, Ylate, and Ym) are: LcTeen-37, LF-1, and ICS-43. These results agree with the GCA values presented in Table 4, for which these three parents have strong negative GCAs while the top parents have positive values.

**Figure 2:**
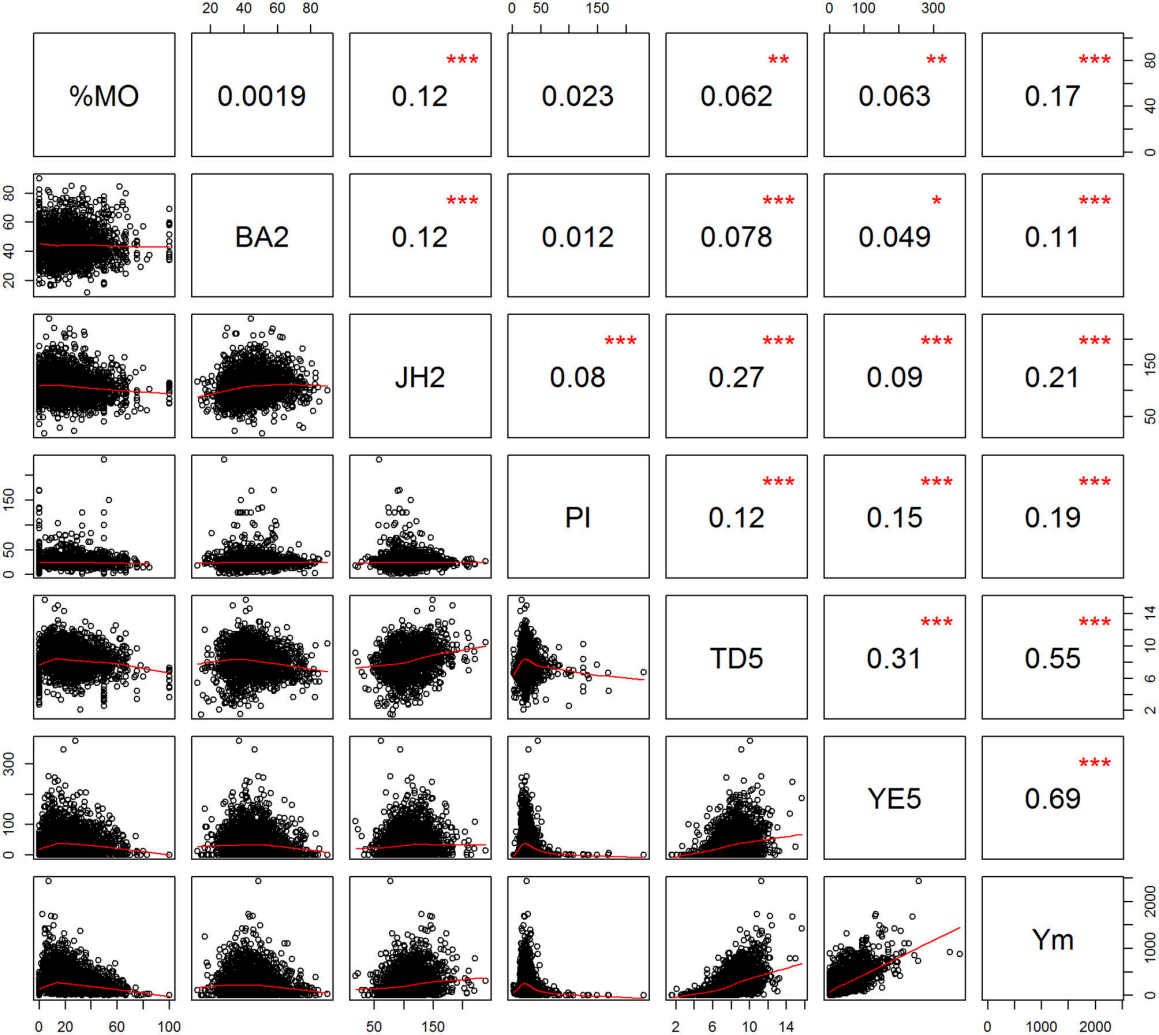
Scatterplot (lower diagonal) and phenotypic Pearson product-moment correlation (upper diagonal) for selected traits. Proportion of Monilia infected pods (%MO), branch angle (BA2), Jorquette height (JH2), pod index (PI), trunk diameter at year five (TD5), yield efficiency at year five (YE5), mean yield ages 2–7 (Ym). Significance levels of are determined by: ***p < 0.001; **p < 0.01; *p < 0.05.

### Ideotype characterization

**Figure 3:**
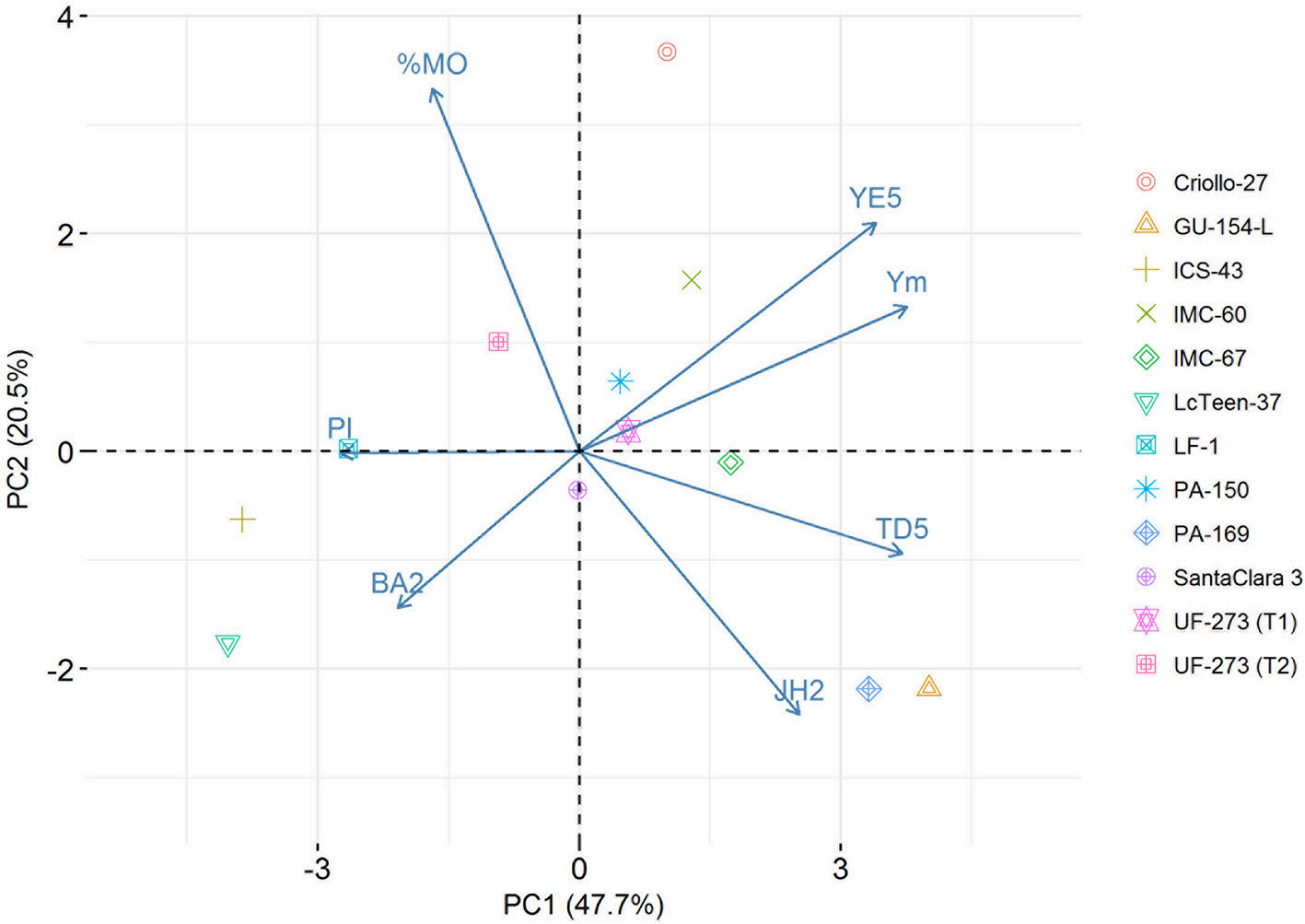
Biplot of selected traits: proportion of Monilia infected pods (%MO), branch angle (BA2), Jorquette height (JH2), pod index (PI), trunk diameter at year five (TD5), yield efficiency at year five (YE5), mean yield ages 2–7 (Ym). The first two PCs of the predicted values for the L7 population account for 68.2% of the total variation. The scores of the parents are overlayed in the plot.

The top 1% best ranked individuals for the weighted index come from only four families: IMC-60 × PA-150, IMC-60 × IMC-67, UF-273 Type1 × IMC-67, and and UF-273 Type 2 × IMC-67 (Supplementary Table S5). For Ym only, the same best families from the combined traits are present with the addition of PA-169 x Criollo-27, PA-169 x GU-154-L, UF-273 Type 1x Criollo-27, and UF-273 Type 2 × GU-154-L (Supplementary Table S10). In terms of agronomic traits, the progenies with low vigor (low TD5) come from families that mainly involved the parents ICS-43 and LcTeen-37, namely, ICS-43 × PA-150, ICS-43 × LcTeen-37, ICS-43 × LF-1, IMC-60 × LcTeen-37, and IMC-60 × PA-150. The only family with relative lower %MO is ICS-43 × LcTeen-37; however, this family ranks low for yield. The best linear unbiased predictions and rankings of single trait shows that only few families provide the best genotypes for a given trait (Table 5). For visual representation of the variation of traits, a biplot encompassing the main yield and vigor characteristics was constructed with the first two principal components (PCs) of the predicted values of the entire population, which accounted for 68.2% of the total variation (Figure 3, Supplementary Table S6). The parents that positively stand out for high Ym are GU-154-L, IMC-60, and Criollo-27. LcTeen-37, LF-1, and ICS-43 are good for reducing trunk diameter, but they also have very low YE5 and Ym. PA-169, GU-154-L, and IMC-67 are related to low %MO, have low PI, and also have moderate to high Ym. Parents with low jorquette height are Criollo-27, ICS-43, Lc-Teen37, UF-273 Type 2, which also have low TD5, these are small trees; however, YE5 is low since these trees have also low average (YM) values and low to moderate PI.

**Table 5:** Weights for selection index ideotype characterization.

**Weight**	**%MO**	**BA2**	**JH2**	**PI**	**TD5**	**YE5**	**Ym**
1	0.05	0.05	0.15	0.15	0.15	0.15	0.30

**Figure 4:**
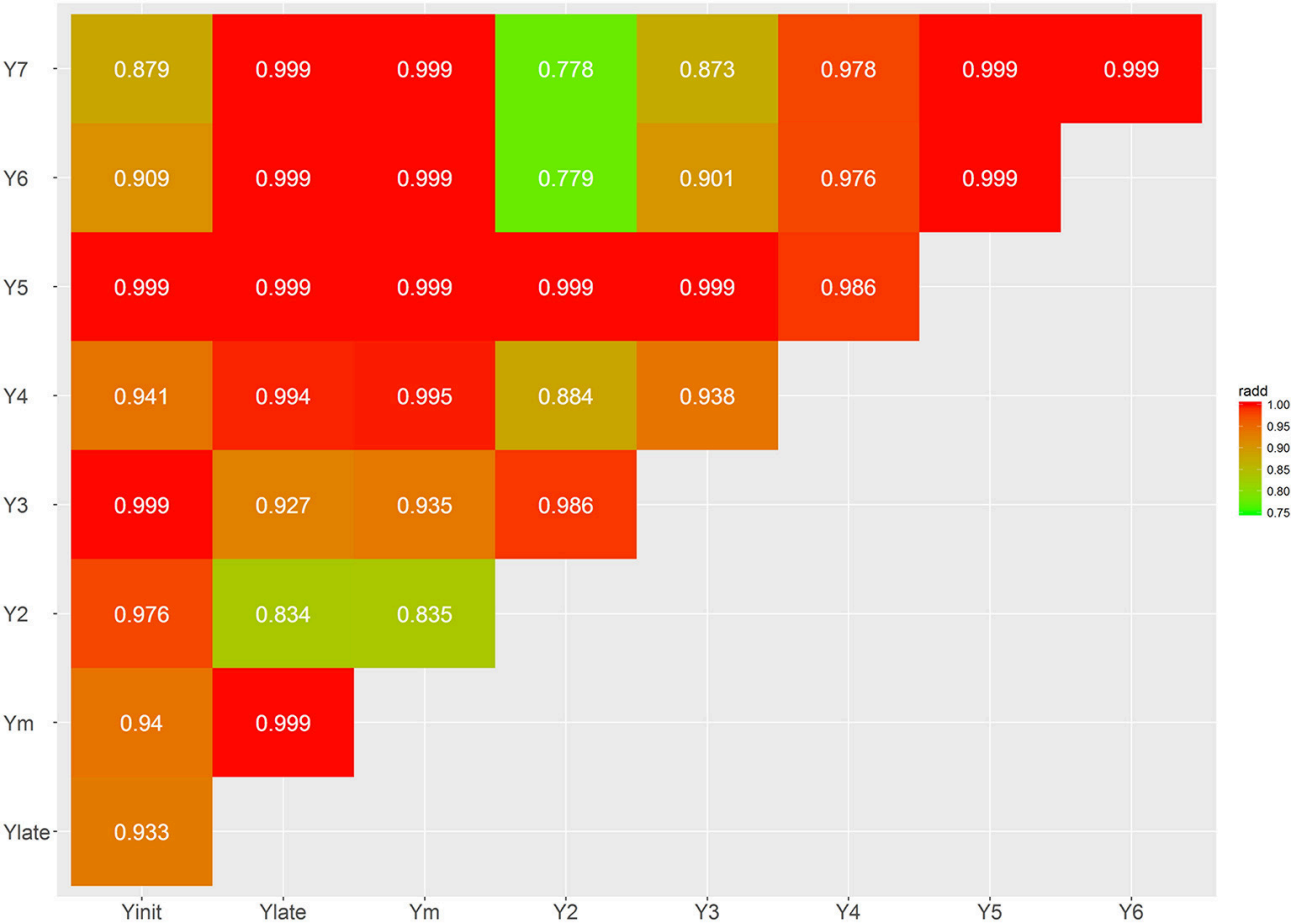
Genetic trait correlations for additive effects: all yield derived traits.

**Figure 5:**
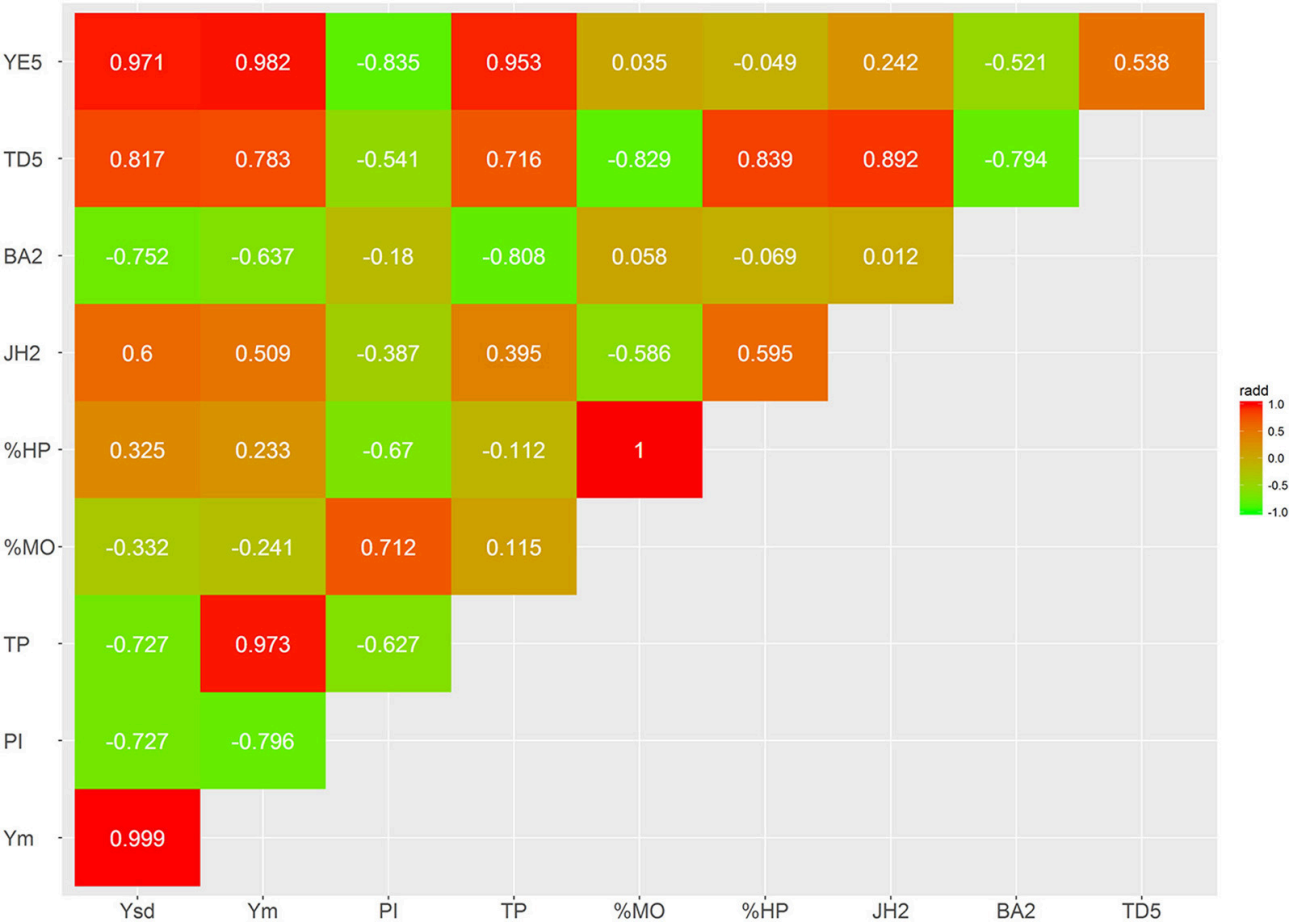
Genetic trait correlations for additive effects: vigor and disease traits.

**Figure 6:**
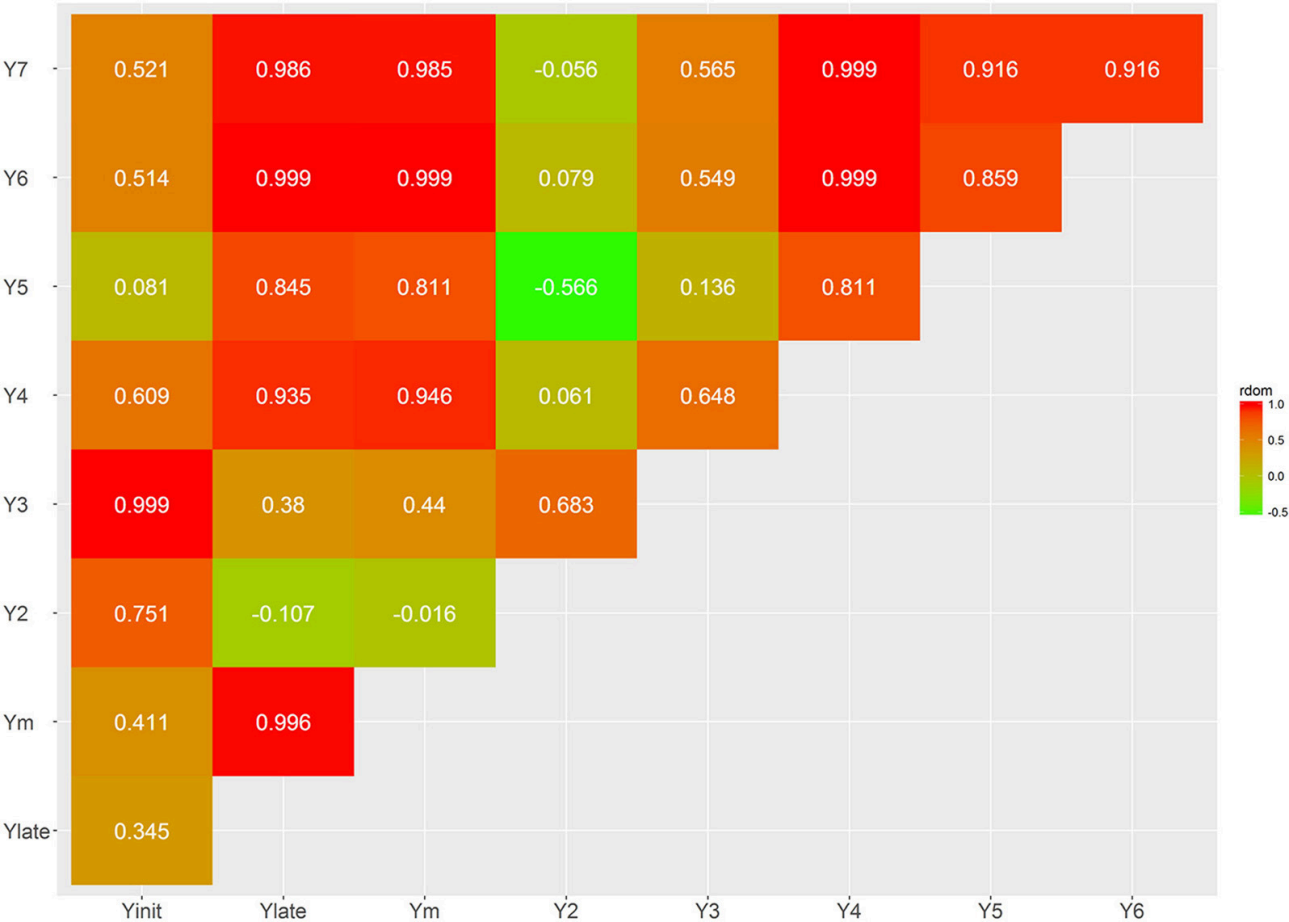
Genetic trait correlations for dominance effects: all yield derived traits.

**Figure 7:**
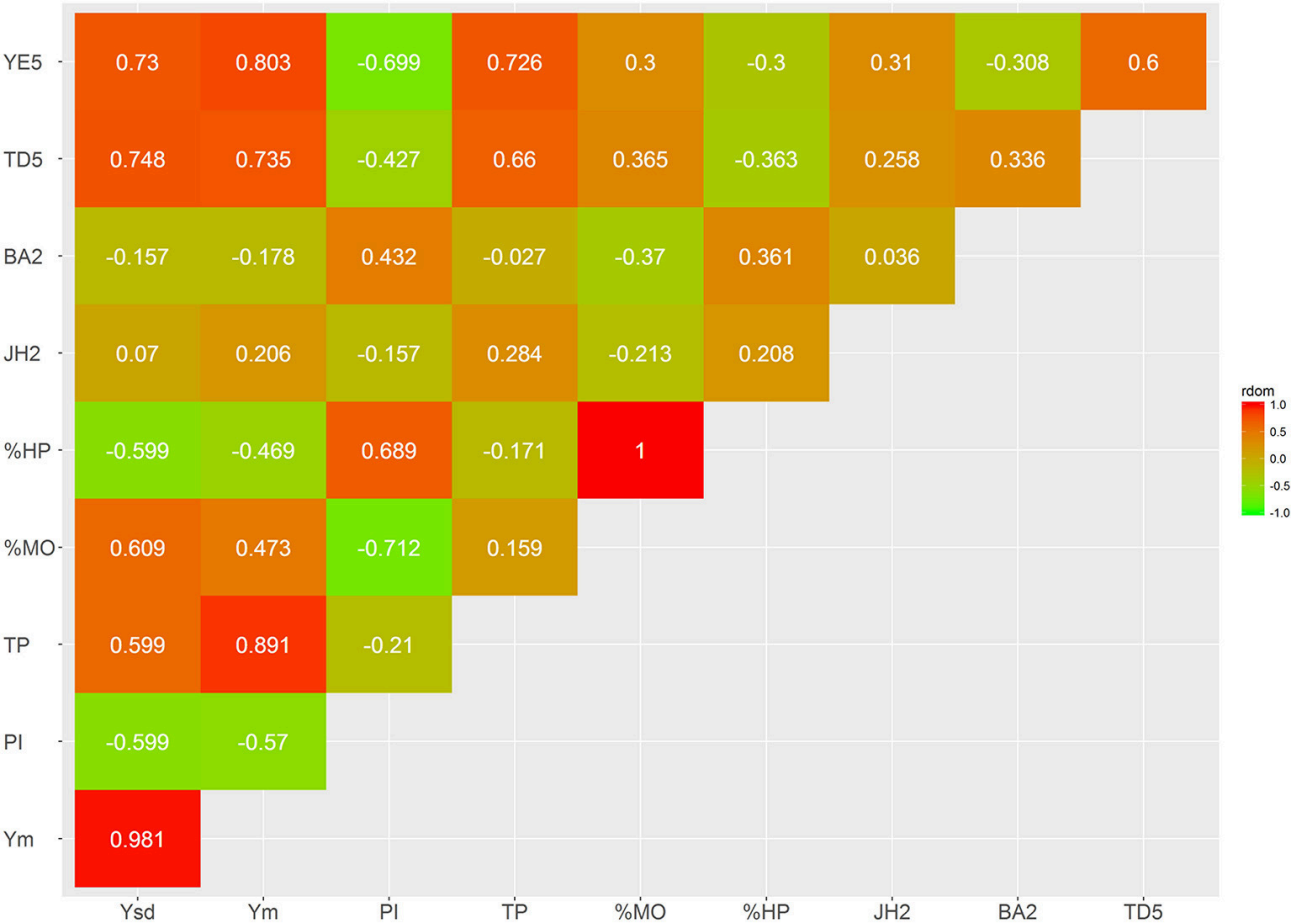
Genetic trait correlations for dominance effects: vigor and disease traits.

### Genetic analyses

The percent heterozygosity for the L10 trial and the corresponding genetic groups for each of the clones is represented in Supplementary Table S2. The L10 clonal trial showed high percentage of heterozygosity across the 96 SNP off-typing markers for most of the clones, with more than 50% heterozygosity for both Santa Clara-3 and ICS43. Santa Clara-3 has 50.9% Contamana, 27.3% Amelonado, and is admixed across the remaining eight genetic groups. ICS43 has 40.8% Amelonado and 51.2% Criollo, with very small contribution from the other genetic groups. The most homozygous clone (2.5% heterozygosity) was GU154L with a 99.1% membership to the Guiana genetic group. Criollo-27 had 41.9% heterozygosity and it is not a pure Criollo, with genetic ancestry belonging to mostly to Amelonado (56.3%) and Criollo (28.2%). In fact, LF1 had the highest Criollo membership (76%) in this population with 28.6% heterozygosity. The two clones with the highest Nacional ancestry are UF273-Type 1 and UF273-Type 2, which are related clones. IMC-60, IMC-67, and PA-150 are mostly Iquitos (98.2, 98, 75%, respectively) and have similar heterozygosity (26.3–29.8%). The most representative clone for the Curaray group was Lc-Teen37, with 40% heterozygosity. PA169 is mainly Marañon (74.4%) and 25.3% heterozygosity. In addition, the Nanay and Purus genetic groups are not represented in the L10 (and thereby) the L7 populations.

## Discussion

A wide range of heritabilities, dominance ratios were estimated from the analysis of the L7 population; which were particularly high for some yield traits, and low for disease traits. In addition, the estimates *h*^2^ and *d*^2^ present important levels of uncertainty (reflected by the magnitude of their standard errors). This is likely due to the small number of parents and families evaluated in this study, in comparison with larger breeding trials, together with the potential presence of off-types in some families. Some traits showed low heritability values, such as %MO, a result that might be due to large environmental and micro-environmental confounding effects in the field. Indeed, the narrow-sense heritability for %MO was low with a value of 0.141 and broad-sense heritability 0.190 (SD 0.075). In our study, we aimed at pyramiding potential different sources of Moniliosis resistant genes by crossing parents with low Moniliosis incidence (PA169, UF273-Type 1) from various cacao genetic groups (Motamayor et al., 2008). This may explain why we did not observe such high levels %MO incidence (we observed a mean of 23% across the years studied, Table 1), relative to the levels reported in farmers' fields (Phillips et al., 2009), and perhaps the lower heritability estimates when compared to other studies. Studies for the same trait, in the same location, reported much higher broad-sense heritabilities: 0.730 (Romero Navarro et al., 2017) and 0.864 with an average incidence of Monilia of 47% (Eugenia and Mu, 2017). Another element that could explain the lower heritabilities for %MO observed is the potential presence of off-type hybrids. In fact, one of the main challenges in cacao breeding programs and other crops is the tendency for mislabeled individuals (off-types; Motilal and Butler, 2003; Turnbull et al., 2004; Hama-Ali et al., 2015; DuVal et al., 2017). Genotyping large populations is not always feasible, due to budget, logistical constraints, and other factors specific to a program. In the case of Romero Navarro et al. (2017), the population studied was adjusted for off-type presence using molecular markers, this approach has significantly improved heritabilities for other diseases in cacao (DuVal et al., 2017).

It is important to note that the heritabilities and dominance ratio estimates for Ym is larger than any of the specific years. This could be due to inconsistent production for a given year and higher proportion of zeros for some individuals, but for total yield over the 6 years, the production becomes more stable as the impact of zeros is dampened (reduced from 80 to 15%).

In most cases, the levels of dominance were similar to the additive component, reflecting large levels of heterosis. Nevertheless, the dominance ratio inflation is likely a result of the diverse and divergent parents in the population. For example, the cross UF-273(T2) × GU-154-L has the largest positive SCA of 0.703 for Ym, where GU-154-L comes from the Guiana structural group and the UF-273(T2) is of Trinitario background, comprised of Amelonado, Criollo, and Nacional genetic groups (Motamayor et al., 2008). We calculated the individual yield of these parents in L10 based on the average number of healthy pods per year (HP) evaluated for tree ages 2–7 (Supplementary Table S11). Similar to total pods (TP), the number of healthy pods (HP) is a trait with high heritability that can be selected for. The trait HP had a heritability of (0.426) which is very similar to that of TP (0.419), since both traits are highly correlated. The HP means from the L10 phenotypes were 1.1 and 4.6 for UF273(T2) and GU-154-L, respectively, with mid-parent value of 2.8. The progeny from this family evaluated in L7 have a mean of 11.7 healthy pods per year. The percent increase of the progenies over the parents (heterosis) is 317 and 154% for the percent increase over the parent with higher HP, GU-154-L. Although a new trial should be established to compare yields between selected grafted seedlings against the grafted parents, these results suggest for the first time the presence of heterobeltiosis in cacao.

Similarly, the SCA for Ym between the clone UF-273(T2) and LcTeen-37, which belongs to the Curaray group, have a negative SCA of (−0.34). As a possible result of diverse backgrounds, the traits PI, TD5, Ysd, TP, and Ylate have a larger dominance ratio than heritability (i.e., *d*^2^/*h*^2^ > 1). Furthermore, a significant positive correlation of 0.39 (*p* = 0.019) was observed between the genetic distance (GD) of the parents and SCA for Ym, which corroborates that heterosis and heterobeltisios occur in cacao perhaps due to allele complementarity between specific diverse backgrounds.

We observed that some crosses involving the highest SCAs in absolute value had one or two parents with high Criollo ancestry, such as ICS43 × LF1, which had an SCA of −0.975 and proportion of Criollo and ancestry of 0.512 and 0.636, respectively for the two parents. ICS43 × IMC67 had an SCA of 0.453, for which IMC67 had 0.257 proportion Criollo. However, UF273T2 × GU154 had the highest positive SCA of 0.703 and they are of Nacional (0.718) and Guiana backgrounds (0.991), respectively.

As described in Cornejo et al. (in review), Criollo ancestry has a negative effect on yield and the Criollo genetic group has a higher level of cumulated deleterious mutations than other genetic groups. However, in this population, the genetic distance is the main predictor of yield. To test the association between Criollo ancestry and SCA for Ym, a generalized linear model was run on SCA as the response variable and the average proportion of the 10 genetic groups between the parents, plus the genetic distance (GD) as covariates. GD had the most significant association to SCA, with an estimate of 0.018 (*p* = 0.00834) but the ancestral groups showed no significance at α = 0.05 in this study.

One of the objectives of this study was to evaluate how yield heritability and dominance effects differ for several evaluations based on measurements that range from 2–7 years of age. The lowest heritability and genetic correlations with any other years was found for Y2. We observed that at age 2, 80% of the trees were unproductive, thereby driving the estimate of heritability to be close to zero. However, there is yield consistency among years, particularly after year three. Although the additive correlations between Yinit (years 2 and 3) and the Ylate (years 4–7) were high (0.933) and it favors early selection of high yielding genotypes; however, Yinit has a lower heritability of 0.352, in contrast to Ylate of 0.542. Hence, even that early selection is possible, by using Yinit, higher precision and certainty will be achieved by concentrating selection on later years by focusing on Ylate. The dominance correlations between these traits was low (0.345). In this case, attention is toward the dominance correlation because the *d*^2^ estimates are high for yield, and large genetic gains come mainly from the strong interactions between parents in this population. The extremes Y2 and Y7, have zero dominance correlation but for Y3 the correlation with Y7 is 0.567, and between Y4 and Y7 it is close to one. Therefore, it is recommended that when trees are at ages 2 and 3, they should not be used for early selection because it has a poor correlation with Ym. At year four, a higher proportion of trees are fully productive, and now this trait is sufficiently informative, and it has a very strong correlation with Ym (0.946).

**Figure 8:**
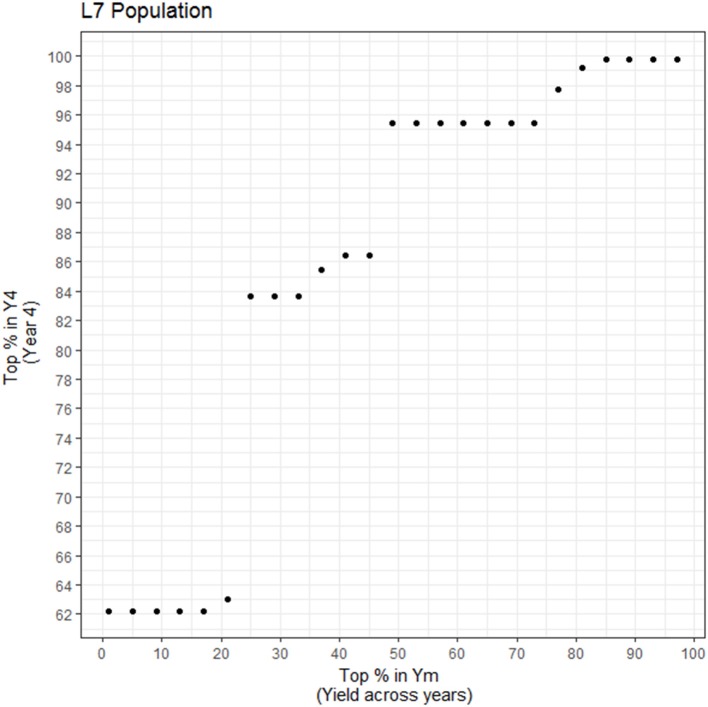
Percentage of the top yielding individuals at year 4 (Y4) needed in order to capture the top percent yielding individuals for average yield (Ym). X-axis: percentage of top individuals taken for Ym; Y-axis. Percentage of Y4 individuals that encompass the selected percentage in Ym (common genotypes).

As mentioned, the first years of measurement (Yinit) do not correlate well with average yield production, Ym (0.411), but at age 4 (Y4), dominance correlations with Ym are high (0.946). If an accelerated breeding approach were to be used by rapidly cloning the best individual hybrid trees, we would then recommend using Y4 as the appropriate age to perform the early selection for yield. In this case, if feasible, we would clone at least the top half at year 4 since the top 10% for Ym is spread within the 62% at Y4 in this population (Figure 8). This calculation may be specific to the population and site studied so, additional studies are recommended before a generalized approach for early yield selection could be recommended. For instance, Figure 8 shows that in order to select ahead of time the top 1–20% trees for Ym, the 62% of the top performers must be selected in Y4. The reason as to why it is the same percentage (62%) for the different selection intensities (between 0 and 20% in Ym) is due to the yield stability of a few genotypes in this population. There are genotypes that did very poorly in Y4 relative to Y5, Y6, and Y7 which are the years that are driving their means higher in Ym. Thus, in order to be able to select them in Ym, the range regarding how many trees must be selected in Y4 is broadened. Trait stability, and in particular, yield stability with respect to time depends on multiple factors, including the effect of genotype by environment interaction, earliness, and alternation (trees with high yields in a season followed by low yields in the next) as it has been found in other crops such as coffee (Cilas et al., 2011). In this study, the trees with largest variability (Ysd) are also the trees that produce the most in terms of average production across years (Ym); These trees have the following characteristics: (1) upward linear trend of increased yield as age of tree increases; (2) increase until a plateau, typically at around year three, then yields drastically drop thereafter and stay low; and (3) increase until a plateau is reached, followed by fluctuating yields thereafter. The second and third points are due to the alternation phenomena that may be due to some genotypes having a plateau and may require more inputs for a physiological reset, greatly affected by the environment, pathogens or pests. Some genotypes are more largely affected by agronomic and environmental conditions than others and perform well in optimal conditions specific to that particular genotype. If the optimal conditions are known for top performing individuals, the alternation in yield can be diminished and higher genetic gains can be achieved (Annicchiarico, 2002).

Many studies focus on examining yield stability in multi-environment trials, however stability over time is also a key trait to consider in a breeding program and such temporal studies are limited in tree crops (Cilas et al., 2011). From our findings, the trait Ysd can be selected for, due to its high heritability (*h*^2^ = 0.459) and also has a strong additive correlation with Ym (0.981), which indicates first, that important improvements can be done by reducing the yield variability on some selected genotypes. However, as we reduce this variability we also reduce the genetic gains for Ym. Here, it is important to select some genotypes that are correlation breakers, but this should be done carefully, as it requires further investigation and understanding of the underlying physiological basis of season to season and year to year productivity variation in cacao as it is known in other perennial crops (Cilas et al., 2011). The selection of genotypes with stable production throughout the years comes at the price of possibly missing the highest potential yields. Farmers may favor stable genotypes, even if those genotypes are not top yielding; or they may favor genotypes that are high yielding only after reaching maturity and larger canopy sizes. In such a case, farmers may consider other ways to generate income until the high yielding trees become productive such intercropping with Banana or other crops.

Total pods, healthy pods, yield, and indirectly, yield efficiency are correlated, as expected. Note that the disease incidence in the study was relatively low, if such is the case, breeding efforts could then focus on selection for total pods in place of measuring the additional yield-related traits.

Large genetic gains are possible for yield-related traits, including Ysd. However, vigor traits have lower genetic gains and are more dependent on the environment as shown by the lower heritabilities obtained for branch angle, trunk diameter and jorquette height. The proportion of healthy and Monilia infected pods also have relatively lower gains (11 and 43.2%, respectively at 5% selection intensity). In regard to increasing genetic gains for disease, improvements can be made by focusing on best parents or families. For example, the strongest GCA effect for %MO comes from PA-169 (−0.338), which belongs to the Marañon structural group. Other parents that are favorable for lowering the effect of proportion of Monilia infected pods in the progeny are GU-154-L and IMC-67. In addition, the family ICS-43 × LF-1 has a favorable interaction (SCA = −0.271). Therefore, selection of the top progenies from the top crosses can improve the genetic gains in disease resistance terms for the next breeding round. However, we observed a low correlation between Ym and %MO despite that no fungicide treatment was applied, therefore, we believe when there's low heritability for disease resistance, that it should have a relative low weight regarding yield when constructing a selection index.

Vigor traits are of importance since they can have an impact in income (less pruning leads to lower labor costs) and total yield per hectare if lower vigor is selected and higher planting densities are used. In this study we found a correlation with high yield and high vigor as has been previously reported (Glendinning, 1966) and also a correlation between high vigor and erect architecture (BA2). Although we found some parents with positive effects on vigor traits, heritabilities for these traits were low. More agronomic research is therefore needed in cacao to decrease vigor via rootstocks as is available for other crops (Lang, 2001; Webster, 2002; Beckman and Lang, 2003). Furthermore, to break the correlation between high vigor and high yield, it would make sense to select for more erect plants that could be planted at higher densities. In this study, we found that trunk diameter (TD5) and yield has a strong positive additive correlation with yield (0.783). One approach to improve yield is to not only break the high vigor-high yield correlation but to also break the correlation between trunk diameter and branch angle which had an additive correlation of −0.794. Larger trunk diameters are correlated with more erect architecture of trees possibly due to branch strength, where stronger branches can more likely support holding a higher angle, while thinner branches are light and have less support, creating a bigger branching angle. Selection of trees with low trunk diameter and high yield and/or trees with low trunk diameter and lower angles would be the correlation breakers that could help improve yields through their tree architecture that allow for higher planting densities. In addition, wood strength would be an interesting trait to evaluate in sub-sequent trials since it may influence branch angle.

**Figure 9:**
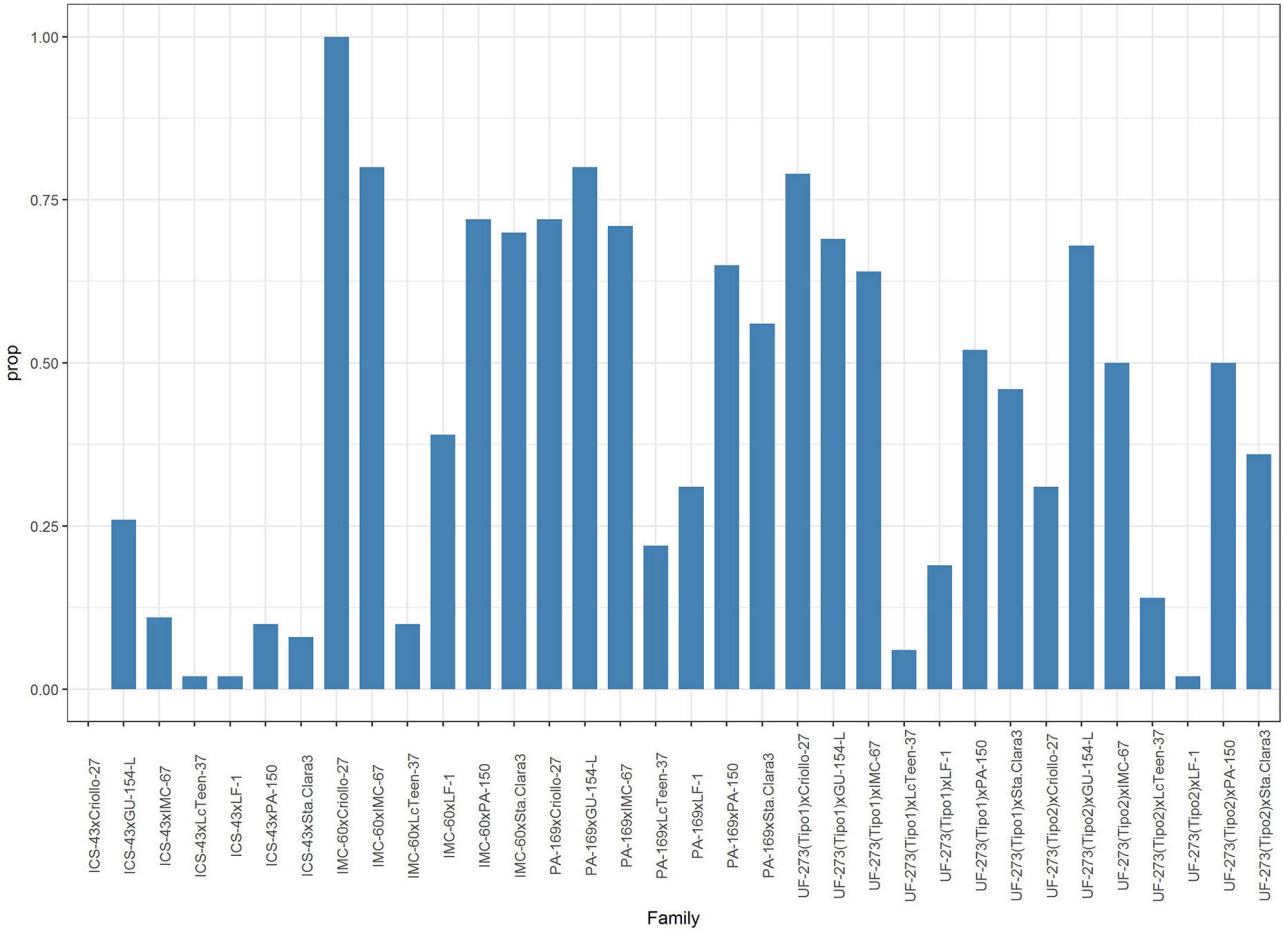
Proportion of individuals within a family greater than the average predicted value of the parents for Ym.

In this study, the L7 population has large dominance effects, and for some traits such as pod index, trunk diameter and yield, it is larger than the additive effects. As previously mentioned, the inflated dominance is likely in part due to the diverse and divergent genetic backgrounds of the parents (Dias, 2001; Motamayor et al., 2008). Often, for any trait that has even moderate to high heritability, a large majority of the top performing individuals tend to come from the same (or few) families (DuVal et al., 2017). For this reason, logically, it would be of best interest to select for families from the best parents instead of individual genotypes. However, given the high heterozygosity of most parents ([Table T2]Supplementary Table S2), the proportion of progenies with values above the average predicted value of the parents is highly variable among families with a range of 0 to 100% and an overall average of 42% for the L7 population (Figure 9). Therefore, it is recommended to test as many as possible divergent parents and to obtain a large number of progenies within the best families. By having large number of progenies within the best families, it would be possible to develop clones from progenies selected with the desired combination of traits. This selection could be feasible by trying to break unfavorable correlations such as high vigor and high yield in the larger families. Interactions are present between the additive effects of the parents and the time frame selected for yield (early vs. late). In Figure 1, it is evident that parents perform differently depending on the time frame for yield. Some parents rank consistently favorably (e.g., GU-154-L, IMC-60, IMC-67) genotypes such as Criollo-27 and PA-169 have a large interaction effect with time frame of evaluation; They both start with a low rank (early yield) and obtain higher ranking for late yield and average yield. In addition, studies that consider crosses between selected progenies would be helpful to isolate additive and dominance effects.

## Author contributions

GM and SG analyzed the data and wrote the manuscript. AA-L collected and analyzed data. AM-Q collected and analyzed data. GM, SG, and JM designed the analysis. WP-M managed collection of field data. JM conceived the experiment, analyzed data and wrote the manuscript.

### Conflict of interest statement

The authors declare that the research was conducted in the absence of any commercial or financial relationships that could be construed as a potential conflict of interest. The reviewer NBMA declared a shared affiliation, with no collaboration,with several of the authors, WP-M, AM-Q, AA-L, to the handling Editor.

## Supplementary material

Supplementary Table S1 Factorial crossing scheme for L7 trial.

Supplementary Table S2 Tree information and heterozygosity of L10 (parents of L7) based on 96 markers spread throughout the genome and percent ancestry for each of the 10 cacao structural groups.

Supplementary Table S3 Bivariate genetic trait correlations for yield, vigor, and disease traits.

Supplementary Table S4 Bivariate genetic trait correlations for yield traits.

Supplementary Table S5 Best linear unbiased predictions and rankings of L7 population according to ideotype.

Supplementary Table S6 Parental Best linear unbiased predictions for traits in Table 1 and the coordinates of the first two PCs.

Supplementary Table S7 Genetic gains based on the percent increase between the average phenotypic values for tree ages 4–7 in the L7 progeny trial, and phenotypic values of the L10 (parental clonal trial) for tree ages 4–9. The traits represented are traits that overlapped between the two trials: TP, HP, MO, PHY, %HP, %MO, and %PHY. The absolute value of genetic gains were taken for disease traits (MO, %MO, and %PHY). Definition of traits are presented in Table 1.

Supplementary Table S8 Genetic gains based on breeding values of selected progenies, overall progenies and parents in the L7 trial. Progenies from the L7 population were ranked from most favorable to least favorable and the average values (i.e., genetic gains) from the top 1% (*n* = 27) and 5% (*n* = 136) performing hybrids trees were calculated. Genetic gains were expressed as the percent increase of the 1% or 5% selection intensities values of the predicted values of progeny over the mean (for the specific trait) from the predicted values of the parents in L7 (*n* = 12). The overall genetic gain (last column) is the entire L7 progeny mean (*n* = 2,720) relative to the L7 predicted parental average (*n* = 12). Traits selected for high values were Y1-Y7, Ynit, Ylate, Ym, HP, YE5 and TP, and traits selected for low values corresponded to Ysd, PI, %MO, JH2 and TD5. Definition of traits are presented in Table 1.

Supplementary Table S9 Genetic gains based on breeding values of selected progenies in the L7 trial. Progenies from the L7 population were ranked from most favorable to least favorable and the average values (i.e., genetic gains) from the top 1% (*n* = 27) and 5% (*n* = 136) performing hybrids trees were calculated. Genetic gains were expressed as the percent increase of the 1 or 5% selection intensities values of the predicted values of progeny over the mean of the average predicted values of the progeny yield for Ym (yield for years 2–7). Definition of traits are presented in Table 1.

Supplementary Table S10 Predicted family values (including missing cross, GU-154 x IMC-60) for yield and vigor traits. The families are ranked by highest yielding (Ym). Definition of traits are presented in Table 1.

Supplementary Table S11 Phenotypic averages for the L10 (parental clonal trial) for ages 4–9.
